# Author Correction: Force-tuned avidity of spike variant-ACE2 interactions viewed on the single-molecule level

**DOI:** 10.1038/s41467-023-36905-2

**Published:** 2023-02-28

**Authors:** Rong Zhu, Daniel Canena, Mateusz Sikora, Miriam Klausberger, Hannah Seferovic, Ahmad Reza Mehdipour, Lisa Hain, Elisabeth Laurent, Vanessa Monteil, Gerald Wirnsberger, Ralph Wieneke, Robert Tampé, Nikolaus F. Kienzl, Lukas Mach, Ali Mirazimi, Yoo Jin Oh, Josef M. Penninger, Gerhard Hummer, Peter Hinterdorfer

**Affiliations:** 1 Department of Experimental Applied Biophysics, Johannes Kepler University Linz, Linz, Austria; 2 Department of Theoretical Biophysics, Max Planck Institute of Biophysics, Frankfurt am Main, Germany; 3 Faculty of Physics, University of Vienna, Vienna, Austria; 4 Malopolska Centre of Biotechnology, Gronostajowa 7A, 30-387 Kraków, Poland; 5 Department of Biotechnology, Institute of Molecular Biotechnology, University of Natural Resources and Life Sciences, Vienna, Vienna, Austria; 6 Center for Molecular Modeling, University of Ghent, Ghent, Belgium; 7 Core Facility Biomolecular & Cellular Analysis, University of Natural Resources and Life Sciences, Vienna, Vienna, Austria; 8 Department of Laboratory Medicine, Unit of Clinical Microbiology, Karolinska Institute and Karolinska University Hospital, Stockholm, Sweden; 9 Apeiron Biologics, Vienna, Austria; 10 Institute of Biochemistry, Biocenter, Goethe University Frankfurt, Frankfurt, Germany; 11 Department of Applied Genetics and Cell Biology, Institute of Plant Biotechnology and Cell Biology, University of Natural Resources and Life Sciences, Vienna, Vienna, Austria; 12 National Veterinary Institute, Uppsala, Sweden; 13 Institute of Molecular Biotechnology of the Austrian Academy of Sciences (IMBA), Vienna, Austria; 14 Department of Medical Genetics, Life Sciences Institute, University of British Columbia, Vancouver, BC Canada; 15 Institute of Biophysics, Goethe University Frankfurt, Frankfurt am Main, Germany

**Keywords:** Single-molecule biophysics, Atomic force microscopy, Computational biophysics

Correction to: *Nature Communications* 10.1038/s41467-022-35641-3, published online 24 December 2022

The original version of this Article incorrectly used nm length unit to describe the opening of SARS-CoV-2 RBD during interaction with ACE2 receptor. Instead of nm, the length distance should be described using Angstroms (Å). This has now been corrected in both the PDF and HTML versions of the Article.

